# Inhibiting complex IL-17A and IL-17RA interactions with a linear peptide

**DOI:** 10.1038/srep26071

**Published:** 2016-05-17

**Authors:** Shenping Liu, Joel Desharnais, Parag V. Sahasrabudhe, Ping Jin, Wei Li, Bryan D. Oates, Suman Shanker, Mary Ellen Banker, Boris A. Chrunyk, Xi Song, Xidong Feng, Matt Griffor, Judith Jimenez, Gang Chen, David Tumelty, Abhijit Bhat, Curt W. Bradshaw, Gary Woodnutt, Rodney W. Lappe, Atli Thorarensen, Xiayang Qiu, Jane M. Withka, Lauren D. Wood

**Affiliations:** 1 Worldwide Research and Development, Pfizer Inc., Eastern Point Road, Groton, CT 06340 USA; 2 Pfizer Worldwide Research and Development, San Diego, CA 92121, USA; 3 Worldwide Research and Development, Pfizer Inc., 610 Main Street, Cambridge, MA 02139, USA; 4 Sanford-Burnham Medical Research Institute, 10901 North Torrey Pines Road, San Diego, CA 92037, USA

## Abstract

IL-17A is a pro-inflammatory cytokine that has been implicated in autoimmune and inflammatory diseases. Monoclonal antibodies inhibiting IL-17A signaling have demonstrated remarkable efficacy, but an oral therapy is still lacking. A high affinity IL-17A peptide antagonist (HAP) of 15 residues was identified through phage-display screening followed by saturation mutagenesis optimization and amino acid substitutions. HAP binds specifically to IL-17A and inhibits the interaction of the cytokine with its receptor, IL-17RA. Tested in primary human cells, HAP blocked the production of multiple inflammatory cytokines. Crystal structure studies revealed that two HAP molecules bind to one IL-17A dimer symmetrically. The N-terminal portions of HAP form a β-strand that inserts between two IL-17A monomers while the C-terminal section forms an α helix that directly blocks IL-17RA from binding to the same region of IL-17A. This mode of inhibition suggests opportunities for developing peptide antagonists against this challenging target.

The family of IL-17 cytokines and receptors consists of six polypeptides, IL-17A-F, and five receptors, IL-17RA-E^1^. IL-17A is secreted from activated Th17 cells, and several innate immune T cell types including macrophages, neutrophils, natural killer cells, and dendritic cells^2^. IL-17A signals through a specific cell surface receptor complex which consists of IL-17RA and IL-17RC^3^. IL-17A’s downstream signaling leads to increased production of inflammatory cytokines such as IL-6, IL-8, CCL-20 and CXCL1 by various mechanisms including stimulation of transcription and stabilization of mRNA^4^,^5^,^6^,^7^. Although various cell types have been reported to express IL-17RA, the highest responses to IL-17A come from epithelial cells, endothelial cells, keratinocytes and fibroblasts^4^.

IL-17A and its signaling is important in host defense against certain fungal and bacterial infections as demonstrated by patients with autoantibodies against IL-17A and IL-17F, or with inborn errors of IL-17 immunity^8^,^9^. In addition to its physiological role, IL-17A is a key pathogenic factor in inflammatory and autoimmune diseases. In phase II and III clinical trials, neutralizing monoclonal antibodies against IL-17A (secukinumab and ixekizumab)^10^,^11^,^12^ or its receptor IL-17RA (brodalumab)^13^ are highly efficacious in treating moderate to severe plaque psoriasis and psoriatic arthritis. Secukinumab has been approved recently as a new psoriasis drug by the US Food and Drug Administration (Cosentyx™)^14^. In addition to psoriasis and psoriatic arthritis, IL-17A blockade has also shown preclinical and clinical efficacies in ankylosing spondylitis and rheumatoid arthritis^15^,^16^,^17^,^18^,^19^,^20^.

Among IL-17 cytokines, IL-17A and IL-17F share the highest homology. These polypeptides form covalent homodimers, and IL-17A and IL-17F also form an IL-17A/IL-17F hetereodimer^21^. Structures are known for apo IL-17F^22^ and its complex with IL-17RA^23^, for apo IL-17A^24^, its complex with an antibody Fab^25^, and its complex with IL-17RA^24^. In these structures, both IL-17A and IL-17F adopt a cysteine-knot fold with two intramolecular disulfides and two interchain disulfide bonds that covalently link two monomers.

There has been active research in identifying orally available chemical entities that would functionally antagonize IL-17A-mediated signaling. Developing small molecules targeting protein-protein interactions is difficult with particular challenges associated with the large, shallow IL-17A/IL-17RA interfaces. Since IL-17RA is a shared receptor for at least IL-17A, IL-17F, IL-17A/IL-17F and IL-17E^21^,^22^,^26^, we chose to seek IL-17A-specific inhibitors that may have more defined pharmacological responses than IL-17RA inhibitors. Our initial approach was to identify peptide inhibitors which could serve as leads for the development of anti-inflammatory therapeutics that could be used alone or in combination with other agents. Our efforts resulted in discovery of a high affinity IL-17A peptide antagonist (HAP), which we attempted to increase the functional production and pharmacokinetics after fusing HAP to antibodies for evaluation as a bispecific therapeutic in animal studies^27^,^28^. Unfortunately, this past work revealed stability issues of the uncapped HAP in cell culture Here, we provide the details of the discovery and optimization that led to HAP and report the complex structure of IL-17A with HAP, which provides structure based rationalization of peptide optimization and structure activity relationship (SAR).

## Results

### Identification of IL-17A peptide inhibitors

Peptides specifically binding to human IL-17A were identified from phage panning using cyclic and linear peptide libraries (Supplementary Figure S1). Positive phage pools were then sub-cloned into a maltose-binding protein (MBP) fusion system. Single clones were isolated and sub-cultured in growth medium, and culture supernatants were used in an enzyme-linked immunosorbent assay (ELISA) to identify specific IL-17A-binding clones. The positive binding supernatants were tested for the ability to block biotinylated IL-17A signaling through IL-17RA in an IL-17A/IL-17RA competition ELISA assay where unlabeled IL-17A was used as positive control to inhibit biotinylated IL-17A binding. Approximately 10% of the clones that specifically bound to IL-17A also prevented the cytokine from binding to IL-17RA. Sequences identified from phage clones were chemically synthesized (Supplementary Table 1) and tested for inhibition of IL-17A binding to IL-17RA (Table 1). A 15-mer linear peptide **1** was shown to block IL-17A/IL-17RA binding with an IC_50_ of 80 nM in the competition ELISA assay (Table 1). This peptide was then tested in a cell-based functional assay wherein production of GRO-α in BJ human fibroblast cells was measured as a function of IL-17A stimulation using 1 ng/ml IL-17A. Peptide **1** was found to be active in this functional assay with an IC_50_ of 370 nM.

### Optimization of IL-17A peptide inhibitors

A SAR campaign was undertaken to improve the potency of peptide **1**. An alanine scan of peptide **2**, an analogue of **1** with a lysine to arginine substitution at position 14, was initiated. When alanine was already present (positions 7 and 15), substitution was made with lysine (Table 1, peptides **3–17**). Positions 1, 2, 4, 5, 7, 14 and 15 were shown to be amenable to substitution without significant loss (less than 3-fold) of binding affinity as measured by the IL-17A/IL-17RA competition ELISA. In particular, at position 5 (**13**), substitution of methionine with alanine resulted in a seven fold improvement in potency (80 nM versus 11 nM respectively). In order to rapidly evaluate the effects of substitution of natural amino acids at tolerant positions identified by the alanine scan, the lead sequence was subjected to site-specific saturation mutagenesis using MBP^29^. Each of the seven positions identified by the alanine scan was individually modified while keeping the rest of the sequence constant. Modifications at positions 2 and 14 were shown to display improvement in binding affinity (data not shown).

Peptides with beneficial point mutations at positions 2, 5, and 14 were synthesized and evaluated in the competition ELISA (Table 1). Two of the changes, V2H (**18**) or V2T (**21**) displayed improved binding in the competition ELISA. Since the replacement of methionine at position 5 with alanine was beneficial, the additional hydrophobic amino acids isoleucine (**24**), leucine (**25**) and valine (**26**) were evaluated and an additional two-three fold improvement in binding was observed for the valine and isoleucine replacements in comparison with alanine. Introduction of a methionine (**27**) or a carboxamide (**28** and **29**) at position 14 was shown to improve the binding affinity of the lead peptide. In general, there was good agreement between the respective binding affinities of the synthesized peptides and their MBP fusion counterparts, except for substitution of valine at position 2 to a tryptophan (**22**), which resulted in a fivefold loss of affinity, for the free peptide when compared with the MBP fusion.

Combining the key amino-acid residues identified by SAR into a single peptide sequence resulted in peptide **30,** named high affinity peptide (HAP), that was found to inhibit IL-17A signaling in a BJ human fibroblast cell assay with an IC_50_ of 17 nM, a more than 20-fold improvement over the phage peptide **1** (Table 2 and Supplementary Figure S2). We also examined the effect of removing the acetyl group at the N-terminus of HAP (which is present in all the peptides made, see Supplementary Material). The un-capped peptide (**31**) had an IC_50_ of 420 nM in the cell-based assay. The loss of cellular activity of **31** was most likely due to the degradation of the N-terminus of **31**, since peptide **31** was shown to be able to bind to IL-17A with similar affinity as HAP itself^27^. Furthermore, our previous work had reported that in antibody fusions the uncapped peptide was degraded under cell assay conditions with removal of the first 1-3 residues to inactive products with the same N-terminal sequences as peptides **32**–**34**^27^. In this work, **32**–**34** are capped by protective acetyl group and reflect the same inactivity as reported. C-terminal truncations showed a more gradual reduction in activity (**35**–**37**; Table 2). After deletion of three amino acids from the C-terminal end (**37**), the peptide is no longer active.

### Dimerization of HAP can further increase its potency

We reasoned that since the IL-17A protein is almost exclusively present in a dimeric form^30^,^31^, dimerizing the IL-17A binding peptides could result in an improvement in binding affinity and inhibitory activity. Homodimers of HAP were made through attachment of polyethylene glycol (PEG) spacers of different lengths at amino acids 4, 7 and 14, as these positions were identified in the alanine scan analysis as not contributing significantly to the activity, and at each N-terminus (Supplementary Table S2). Due to the high reactivity of the pentafluoroester (PFP) group used as the activating group in the PEG, the histidine at position 2 and the lysine at position 15 were replaced with threonine and dimethyllysine respectively to prevent formation of side products, which resulted in peptide **38** that was comparable in activity with HAP. This exercise revealed that several dimeric peptides with the longer PEG21 spacer were significantly more potent than the monomer peptide in the cell-based assay (Supplementary Table S2). Peptide **45**, dimerized via attachment of a PEG21 spacer at position 14 (Supplementary Scheme S1 and Figure S3), was the most potent with cellular IC_50_ of 0.1 nM. This significant improvement in antagonism was not seen in the peptide monomer functionalized with a PEG21 group at position 14 as peptide **48** had an IC_50_ of 21 nM (Supplementary Scheme S2).

The species cross-reactivity of the dimeric peptide **45** and HAP were assessed in a murine functional cell assay using 15 ng/ml murine IL-17A. Peptide **45** blocked the receptor binding of murine IL-17A although with potency two orders of magnitude weaker than that observed against human IL-17A (IC_50_ = 41 nM vs IC_50_ = 0.1 nM, respectively). The monomer HAP was much weaker (IC_50_ >1 μM) in inhibiting murine IL-17A signaling (Supplementary Figure S4).

Although the dimeric peptide **45** is much more potent than HAP in the cell-based assay, in subsequent studies we decided to focus our efforts solely on characterizations of the monomeric peptide HAP in hopes to identify smaller peptide inhibitors containing the best minimal functional group.

### Orthogonal assays to confirm HAP antagonism

**Figure 1:**
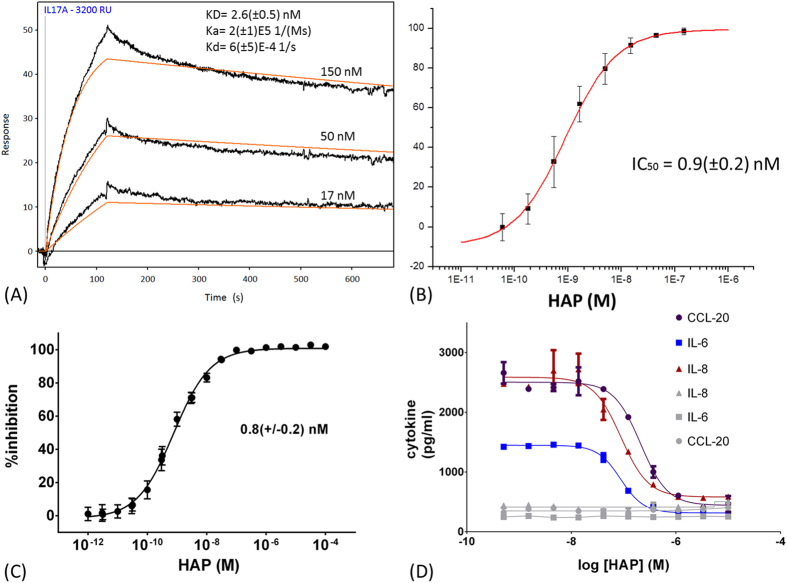
Binding of HAP to IL-17A and inhibition of IL-17A/IL-17RA are measured by SPR, FRET and cell-based assays. (**A**) Typical SPR sensorgrams (black) of HAP at indicated concentrations binding to biotinylated human IL-17A immobilized on a streptavidin chip surface, fitted with single site binding model curves (red). Kinetic parameters (k_a_, k_d_) were obtained by a global fit using three concentrations in triplicate. K_D_ determined by the standard equation, K_D_ = k_d_/k_a_. (**B**) HAP inhibits SPR signaling of IL-17A binding to immobilized IL-17RA. Data are mean and error bars of +/− standard deviation of three measurements. (**C**) Inhibition of IL-17A and IL-17RA binding by HAP measured by FRET assay. Data are mean and error bars of +/− standard deviation from 299 experiments, each performed in duplicate. (**D**) Example of HAP selective inhibition of the production of IL-8 (triangles), IL-6 (squares) and CCL-20 (circles) by primary human keratinocyte cells synergistically stimulated by 100 ng/ml IL-17A and 10 ng/ml TNF-α. HAP does not inhibit the baseline production of IL-6, IL-8 and CCL-20 stimulated by 10 ng/ml TNF-α alone (gray lines and symbols). Data are mean and error bars of +/− standard deviation of duplicated experiments.

To further characterize the interaction of HAP with IL-17A, we set out to determine its *in vitro* binding affinity, specificity and kinetic profile using Surface Plasmon Resonance (SPR) methods (Fig. 1A). HAP binds to immobilized human IL-17A homodimer tightly (Table 3). It has slightly weaker affinity for human IL-17A/F heterodimer and >10 fold weaker affinity for mouse IL-17A (Table 3). HAP does not show significant binding to immobilized human IL-17F homodimer or IL-17RA at concentrations up to 100 nM.

Additionally, we investigated the antagonism of the human IL-17A/IL-17RA interaction by HAP using orthogonal methods including SPR and Förster resonance energy transfer (FRET) competition assays (Fig. 1B,C). In both assays, incubation of IL-17A with HAP effectively blocks the binding of IL-17A to immobilized IL-17RA with similar sub-nM IC_50_ (Table 3).

### HAP blocks IL-17A signaling in a human primary cell assay

While either IL-17A or TNF-α alone can stimulate the release of multiple inflammatory cytokines, when acting together they can synergistically enhance each other’s effects (Supplementary Figure S5). These integrative responses to IL-17A and TNF-α in human keratinocytes have been reported to account for key inflammatory pathogenic circuits in psoriasis^32^. Thus, we chose to study HAP’s efficacy in blocking the production of IL-8, IL-6 and CCL-20 by primary human keratinocytes stimulated by IL-17A in the presence of TNF-α, an assay which may be more disease-relevant. HAP inhibits the production of all three cytokines in a dose-dependent fashion (Fig. 1D). Significantly, the baseline levels of IL-8, IL-6 and CCL-20 stimulated by TNF-α alone are not inhibited by HAP, further indicating the selectivity of HAP (Fig. 1D). Such pharmacological selectivity may be important to suppress inflammatory pathogenic circuits in psoriasis, while sparing the anti-infectious immune responses produced by TNF-α. The relatively high IC_50_ values in this assay (Table 3) are probably due to the high IL-17A concentration (100 ng/ml) needed for detection of IL-6. As a reference, a commercial anti-IL-17A antibody (R&D Systems) inhibits the production of IL-8 with an IC50 of 13(±6) nM (N = 3). Indeed, the IC_50_ was 14(±9) nM (N = 12) for HAP inhibition of IL-8 production when only 5 ng/ml IL-17A was used in this assay. In patients, the concentration of IL-17A in psoriatic lesions is reported to be 0.01 ng/ml, well below the EC_50_ (5–10ng/ml) of IL-17A induced IL-8 production *in vitro*^3^,^33^.

Similar to keratinocytes assay results, while HAP inhibits IL-17A stimulated IL-6 production by BJ human fibroblast potently (IC_50_ of 17 nM), it does not inhibit TNF-α stimulated IL-6 production at concentrations up to 10 μM (Supplementary Figure S2).

### Crystallization and structure determination

Extensive crystallization trials, either by co-crystallization or by soaking HAP into preformed apo IL-17A crystals^24^, failed to lead to an IL-17A/HAP complex crystals. We theorized that HAP binding induced large conformational changes in IL-17A that led to the difficulty of getting an IL-17A/HAP binary complex crystal. It is known that an antibody antigen-binding fragment (Fab) can be used as crystallization chaperones in crystallizing difficult targets^34^. We hypothesized that HAP may target the N-terminal of IL-17A which is known to be more flexible than its C-terminal^24^,^25^ and conformational changes needed for HAP binding may be more likely there. We designed an antibody Fab known to target the C-terminal half of IL-17A based on a published IL-17A/Fab complex crystal structure^25^, and produced it in HEK293 cells. In an SPR assay HAP and this Fab were able to co-bind IL-17A without large changes in their binding affinities and kinetics, confirming our hypothesis (Supplementary Figure S6). Furthermore, since it binds to an area far away from that of HAP (see below), this Fab should have minimum effects on HAP binding conformation. Crystals of Fab/IL-17A/HAP ternary complex were obtained readily in crystallization screens.

Crystallization of IL-17A and its binding partners was accomplished using two forms of IL-17A. These were, respectively, a presumably more homogeneous form of IL-17A that lacked the disordered N-terminal peptide and a full-length form of the cytokine with a full complement of disulfide bonds. (see Method). Crystals of the Fab/truncated IL-17A/HAP complex diffracted to 2.2 Å, and the Fab/full length IL-17A/HAP complex diffracted to 3.0 Å (Supplementary Table S3). Both structures were solved by molecular replacement. Both complexes crystallized in the space group of P321, with half the complex (1 Fab/1 IL-17A monomer/1 HAP) in the asymmetric unit. The intact complex can be generated by applying crystallographic 2-fold symmetry. Electron densities for HAP residues Ile1-Asn14 were readily interpretable with the exception of Lys15, which is disordered. When considering the protein, the complex structure containing the full length IL-17A is identical to that of the truncated IL-17A, with the exception of Cys106 (Ser106 in the truncated IL-17A), which is disordered. Cys106 is covalently linked to Cys10 that resides in the disordered N-terminal peptide in the full length IL-17A.

### Overall structure of Fab/IL-17A/HAP complex

**Figure 2:**
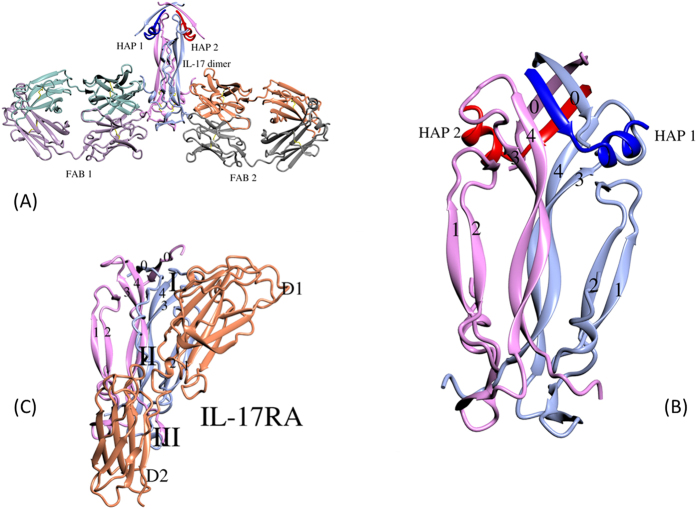
Overall structure of the Fab/IL-17A/HAP complex in ribbon presentation. For clarity, different molecules are colored differently. Two HAP molecules are colored blue and red, and IL-17A monomers are colored ice blue and pink, respectively. Picture prepared using program CCP4MG^46^. (**A**) Overview of the distinct binding sites of Fab and HAP to IL-17A. (**B**) Close-in view of the IL-17A/HAP structure. IL-17A β-strands are labelled. Each of the two bound HAP interacts with both monomers of the IL-17A dimer. (**C**) As a comparison, the IL-17A/IL-17RA complex was shown with IL-17A in the same orientation. Three distinct areas IL-17A/IL-17RA interface are labeled.

**Figure 3:**
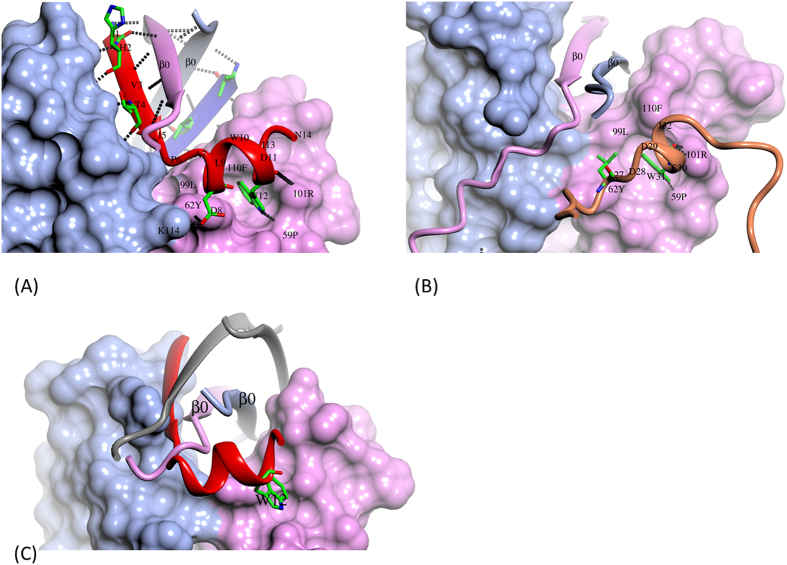
Mechanism of the inhibition of the IL-17A/IL-17RA interaction by HAP. (**A**) HAP binds at region I of IL-17A. IL-17A dimer is in surface presentation (β-strands 0 shown as ribbons for clarity). Polar interactions are shown in dashes. HAP residues as well as key IL-17A residues are labeled. For clarity, a few HAP residues are also shown in stick model with carbon atoms colored green, oxygen in red and nitrogen in blue. (**B**) I-17RA (ribbon in gold) peptide Leu27-Ile32 binds to the same area as the HAP α-helix. Trp31 of IL-17RA binds to the same pocket in IL-17A as Trp12 of HAP. (**C**) As illustrated by overlay a single HAP molecule and β-strands 0 (grey) of the IL-17A/HAP complex in the apo IL-17A structure, conformational changes in region I of IL-17A are needed for binding of both the β-stand and α-helix of the HAP. Notice that the Trp binding pocket for W12 of HAP or W31 of IL-17RA is missing in the apo structure.

In a similar manner to the published structure of Fab/IL-17A complex^25^, two Fab molecules bind symmetrically to the C-terminal of the cytokine dimer, interacting with epitopes from both monomers (Fig. 2A). Two copies of HAP bind to the N-terminal of the cytokine dimer, also symmetrically, and each HAP molecule also interacts with both IL-17A monomers (Fig. 2). Based on disclosed epitopes of Secukinumab and Ixekizumab^35^,^36^, HAP binds to IL-17A at an area that is also different from those of those two antibodies. The N-terminal 5 residues of HAP, ^1^IHVTI, form an amphipathic β-strand that inserts between β-strand 4 of one IL-17A monomer and β-strand 0 (the first ordered peptide of IL-17A) of the second monomer. This β-strand is parallel to both strands 0 and 4 (Fig. 3B). Strands 0 of two IL-17A monomer are antiparallel, as appeared in other IL-17A structures^24^. The C-terminal 8 residues of the HAP that are ordered in the structure, ^7^ADLWDWIN, form an amphipathic α-helix interacting with the second IL-17A monomer. Pro6 of HAP makes a transition between the N-terminal β-strand and the C-terminal α-helix of HAP. As a comparison, an IL-17A/IL-17RA complex structure (PDB code 4HSA) is also shown with IL-17A in the same orientation (Fig. 2C).

### Inhibition mechanism of IL-17A signaling by HAP

IL-17RA binds IL-17A at three regions on the IL-17A homodimer^24^. HAP binds IL-17A at region I. Region I is formed by residues at the ends of β strands 0 and 4, and from loops 1–2 and 3–4 of IL-17A (Fig. 2). Conformational changes in region I induced by HAP binding alone may allosterically affect IL-17RA binding, but more importantly, the α-helix of HAP directly competes with IL-17RA for binding to IL-17A (Fig. 3). The most significant interactions between the α helix of HAP and IL-17A involve Trp12 of HAP, which binds in a hydrophobic pocket in IL-17A formed by the side chains of Phe110, Tyr62, Pro59 and the hydrophobic portion of the Arg101 side chain (Fig. 3A). The Trp12 side chain of HAP donates a hydrogen bond to the main chain oxygen of Pro69 of IL-17A. The positively charged Arg101 side chain of the IL-17A engages in a charge-helix dipole interaction with the main chain oxygen of Trp12. Additionally, Leu9 and Ile13 of the HAP have hydrophobic interactions with IL-17A, and the Asp8 side chain has hydrogen bond and ion pair interactions with Tyr62 and Lys114 of IL-17A, respectively.

In region I, an IL-17RA peptide interacts with IL-17A in a very similar fashion to the α-helix of HAP. The IL-17RA peptide has sequences of ^27^LDDSWI, and part of the peptide is also α-helical (Fig. 3B). Leu7, Trp31 and Ile32 of IL-17RA interact very similarly with the same residues of IL-17A as Leu9, Trp12 and Ile13 of HAP (Fig. 3B). In this sense, the α-helix of HAP with a sequence of ^9^LWDWI is a good mimetic of the ^27^LDDSWI peptide of IL-17RA.

The β-strand of HAP has no equivalent in IL-17RA. However, it mimics the β-strand 0 of IL-17A. The amphipathic β-strand of HAP orients the hydrophilic side chains of His2 and Thr4 outwards, and the hydrophobic side chains of Ile1, Val3 and Ile5 inward (Fig. 3A). β-strand 0 in IL-17A is also amphipathic with the sequence of ^21^TVMVNLNI. In all IL-17A structures obtained to date, β-strand 0 orients the hydrophilic side chains of Thr21, Asn25 and Asn27 outward, and the hydrophobic side chains of Val22, Val24, Leu26 and Ile28 inward.

The binding pocket occupied by either Trp12 of HAP or Trp31 of IL-17RA is not formed in the apo IL-17A structure (Fig. 3C). Conformational changes of IL-17A are needed for both HAP and IL-17RA to bind to that region. Particularly for HAP, β-strands 0 have to shift out of the hydrophobic cleft formed by the main body of the IL-17A by as much as 10 Å between Cα atoms (Fig. 3C). Disruptions of the apo IL-17A structure by HAP binding are apparently compensated for by formation of the new interactions that involve almost the entire HAP molecule (Fig. 3B).

### Structure basis for the observed SAR of peptides

The IL-17A/HAP complex structure obtained is very consistent with the observed SAR of our identified peptide inhibitors, explaining well how the evolution of the initial phage peptide **1** to HAP and **45** improved its potency (Supplementary Figure S7). The important interactions involving Trp12 of HAP explain the >90 times drop in potency of the W12A variant (**6** vs **1**, Table 1). The amphipathic nature of the HAP β-strand explains the preference of the hydrophilic residues at the 2 and 4 positions of peptides (**14**, **18**, **19**, **21** and **23** vs **1** and **22**, Table 1). All N-terminal residues of HAP are part of the β-sheet with β-stands 0 and 4 of IL-17A, which explains why removal of the first 1–3 residues completely abolishes the ability of HAP to block IL-17A cell signaling (**31**,**32** and **33**, Table 2). The C-terminal Asn14 and Lys15 of HAP are not directly involved in interactions with IL-17A, and this is reflected in the gradual reduction in activity caused by C-terminal truncations (**35** and **36**, Table 2). Each peptide monomer in **45** may not necessarily be more potent than HAP, but two monomer peptides within the same molecule that can simultaneously bind to IL-17A can greatly improve its potency due to avidity effects.

HAP targets region I of IL-17A, an area that has the least sequence conservation in IL-17 cytokines^22^,^24^. This lack of sequence conservation in the HAP binding site explains the observed specificity of HAP binding to human IL-17A. For example, inspection of the published IL-17F crystal structure (PDB code 1JPY) revealed a pocket of IL-17F similar to that of IL-17A for W12 of HAP binding, but it is occupied by a Phe-Phe motif at the N-terminal peptide of IL-17F. This Phe-Phe motif is missing in IL-17A. Sequence alignments between human and mouse IL-17A^24^ indicated that among IL-17A residues that interacting with HAP, majority differences occur in strand 0 of IL-17A which interacts with the N-terminal β-strand of HAP. In human IL-17A the sequences are ^21^TVMVNLNI, and in mouse they are ^21^NVKVNLKV.

## Discussion

Using a combination of phage display and SAR we have discovered novel peptides that are IL-17A antagonists. One of those peptides, HAP, also shows activity in inhibiting the production of multiple inflammatory cytokines by primary human keratinocytes stimulated by IL-17A and TNF-α, a disease relevant-model. We have also determined the complex structure of IL-17A/HAP, which provides the structural basis for HAP’s antagonism to IL-17A signaling.

During IL-17A signaling, IL-17A binds to one copy of IL-17RA and one copy of IL-17RC^21^,^23^,^24^. Since apo IL-17A is a homodimer with 2 fold symmetry, IL-17RA potentially can bind to either face of the IL-17A dimer. With two HAP molecules covering both faces of the IL-17A dimer, HAP can block IL-17RA approaching from either face. To form the 1:2 complex observed in crystal structure, it is important that there is no strong negative cooperativity in the binding of two HAP molecules. In fact, in native electrospray ionization mass spectrometry analysis only 1:2 IL-17A/HAP complex was observed even when IL-17A was in excess (Supplementary Figure S8), indicating a positive binding cooperativity that favors inhibition of IL-17RA binding by HAP.

HAP, with only 15 residues, can achieve almost the same binding affinity as the much larger IL-17RA molecule, indicating a more efficient way of binding to IL-17A. The interaction of IL-17A with IL-17RA has an extensive interface, covering ~2,200 Å^2^ surface area of IL-17A^24^. Due to the discontinuous nature of the IL-17A/IL-17RA binding interface, it is classified as having tertiary structural epitopes on both binding partners, and is therefore hard to target using small molecules^37^. Our studies of HAP demonstrated an uncommon mode of action for a peptide in inhibiting such a difficult protein-protein interaction target, and suggest further possible improvements in its binding potency.

One way of further improving HAP’s potency is by dimerization. Homo-dimerization of HAP (**45)** achieved sub-nanomolar potency against human IL-17A in cell assay. In the crystal structure, the distance between the carbonyl of Asn14 of one HAP molecule and the N-terminus of the second is only 15.7 Å, suggesting the potential for more potent dimeric peptides to be designed by using linkers of different lengths at different positions.

Another direction of improving HAP is by reducing its size. As demonstrated by the crystal structure, binding of the α-helix of HAP should be sufficient for preventing IL-17RA binding to IL-17A. Theoretically, it is possible to design chemicals such as stapled α-helical peptides to block α-helix-mediated IL-17A/IL-17RA interactions. Such peptides may have smaller sizes with more favorable physical properties (proteolytic resistance, serum half-life, permeability, etc)^38^,^39^,^40^.

In summary, these peptide-based anti-IL-17A modalities could be further developed as alternative therapeutic options to the reported monoclonal antibodies. We are also very interested in finding non-peptidic small molecule IL-17A antagonists, and HAP can be used as an excellent tool peptide. The strategy utilized in generating the complex structures of HAP may also be useful for enabling structure based design of some known small molecule IL-17A antagonists^41^.

## Methods

### Peptide synthesis

Peptides were synthesized either on a PTI Symphony or Biotage Syro II synthesizer employing standard Fmoc chemistry on Rink resin with N-terminal amine capped with acetyl group unless noted otherwise. Resin, amino acids and solvents were purchased from EMD Chemicals. Peptides were then removed from polymer support using the cleavage cocktail (88:5:5:2, TFA:PhOH:H_2_O:TIPS) and precipitated with cold diethyl ether. For the dimers, the native peptides incorporating a primary amine (N-terminal amine or lysine at specific positions in the peptide sequence) were mixed with the bis-pentafluoroesters of the desired PEG (purchased from Quanta BioDesign) in the presence of DIEA in DMF. Peptides were purified by reversed phase HPLC on Phenomenex preparative Luna C18 columns using water:acetonitrile gradients and lyophilized.

### HPLC-MS conditions for peptide purity analysis

All peptides were assessed for purity by analytical C18 RP-HP-LCMS prior to use in biological assays. Buffers used were 0.1% trifluoroacetic acid in water (A) and 0.1% trifluoroacetic acid in acetonitrile (B). The standard method (1) consisted of a linear gradient of 5% to 95% B over 10 minutes on Agilent 1100 series HPLC-MSD and method (2) consisted of a linear gradient of 5% to 95% B over 8 minutes on Waters 2767 series HPLC-MSD. The C18 column (Phenomenex, Luna C18, 4.6 × 150mm) effluent was immediately mass analyzed in electrospray positive mode. Accurate mass measurements of final peptides were performed using C18 reversed-phase chromatography mass spectrometry (RPHPLCMS) and mass detected on a Waters Synapt G2 Q-Tof mass spectrometer tuned to a resolution (FWHM) of 25,000. Exact intact masses were calculated based on the monoisotopic m/z value of the base peak charge state. All peptides were analyzed using these methods. Supporting data of peptide **45** is shown (Supplementary Figure S2) as representative data sets of the all the molecules investigated (characterization of all peptides presented on Table 1S).

### ELISA assay

rhIL-17 (#317-ILB), IL-17R (#2269-IL) were from R&D Systems. ELISA wash buffer (50-63-01) and TMB SureBlue Microwell Peroxidase substrate (#50-63-01 or #52-00-00) were from KPL. Half-well high-binding ELISA plates (#3690) and full-well plates (#9018) were from Costar. Superblock (#2011 or AAA500) was from ScyTek. No-Weigh NSH-PEG4-Biotin (21329) and Zeba Desalt Spin Columns (89882) were from Pierce.

ELISAs were as follows in duplicate unless otherwise specified. Plates were coated in PBS overnight at 4 °C and blocked using Superblock for one hour at RT. Subsequent incubations were for 1 hr at room temperature with dilutions in Superblock. Plates were washed 3X between steps using a Biotek ELx405 plate washer and developed using TMB substrate. Reactions were stopped in 2 M H_2_SO_4_ and OD_450_ values were read on a Molecular Devices SpectraMax Plus plate reader.

IL-17 RA competition: half-well plates were coated with 0.5 μg/mL IL-17R-Fc and blocked. Peptides were titrated in Superblock containing 0.4 μg/mL Biotinylated IL-17A. Biotinylated IL-17A was detected by adding tetramethylbenzidine (TMB). Reactions were stopped in 2 M H2SO4 and OD450 values were read on a Molecular Devices SpectraMax Plus plate reader.

### Human BJ fibroblast and murine MLE-12 cell assays for inhibitor screen

For inhibitor screen, human BJ fibroblast cells (ATCC CRL2522) (American Type Culture Collection, VA) were used. For mouse cell based assay MLE-12 mouse epithelial cells (ATCC CRL2110) were used. Both cell lines were maintained in ATCC recommended media. Cells were seeded at 5 × 10^3^ cells/well into 96-well flat-bottom microtiter plates in which peptides that had been pre-diluted with cytokines (1 ng/mL for human IL-17A or 15 ng/mL for mouse IL-17A) in culture medium. Cells were incubated at 37 °C for 16–24 hrs, and supernatants were collected and analyzed by ELISA for either human CXCL1/GRO-α (R&D Systems DY275) or mouse CXCL1/KC (R&D Systems DY453).

### Primary human keratinocytes cell assay

Primary human keratinocytes were cultured in Epilife medium with EDGS (Life Technology, Cascade Biologics) following product instructions. 5 days after establishing the culture from frozen vials, cells were plated at 10,000/well (80 μl) in culture media in 384 well plate. 4 hours after plating the cells, 10 μl of 10X peptide stocks were added. Final DMSO concentration was 1%. Immediately after peptide addition, 10 μl of a mixture of recombinant human IL-17A (endotoxin Level <0.10 EU per 1 μg of the protein, E.coli expression, >97% pure judged by SDS/page, R&D System, Minneapolis, MN) 100 ng /ml (final) and TNF-α (Sigma-Aldrich, St Louis, MO) 10 ng/ ml (final), or TNF-α 10 ng/ml only were added to the cells. Cell assay plates were incubated for 48 hours at 37 °C in a tissue culture incubator before harvesting culture supernatants for analysis of IL-6, IL-8 and CCL-20 production using kits K211AKB-2, K211ANB-2, and K211BEB-2, respectively (Meso Scale Discovery, Rockville, MD).

### Protein production

Details of non-commercial protein constructs used in this study and their purifications are in the Supplementary Material.

### SPR binding assays

The IL-17 SPR binding assay was run on a Biacore 3000 SPR instrument (GE Healthcare). Biotinylated human IL-17A, or IL-17A/F heteromer, or IL-17F, or mouse IL-17A (Cell Signaling) was captured on a Biacore Streptavidin chip to achieve protein density of about 2500 to 3500 RUs on the surface. The SPR running buffer was 10 mM HEPES, pH 7.4, 150 mM NaCl, 0.01% P20 with 3% DMSO. Peptide samples were injected at a flow rate of 50 μl/min for 180 seconds association and at least 600 seconds dissociation using a 2–3 fold dilution series. Multiple blank injections were run before and after each peptide series for references. The data were processed and analyzed with Scrubber 2.0 (BioLogical Software) and Biaeval software (GE Healthcare) to calculate binding constants and on and off rates.

The IL-17A SPR competition assay was run on a BioRAD ProteON instrument (BioRAD Laboratories). IL-17RA-Fc fusion protein (R&D Systems) was captured on a GLM chip using the standard amine coupling reaction. The chip surface activated using a mixture of sulfo-NHS and EDC, was exposed to the receptor dissolved in acetate buffer pH 5.0 at 0.01 mg/ml concentration. The surface was deactivated using 1 M ethanolamine HCl. An adjacent flow cell on the chip was treated identically without the protein and was used as a reference surface for subsequent data analysis. Three-fold dilution series of peptides mixed with 5 nM IL-17A were injected at a flow rate of 50 μl/min for 120 seconds, followed by 300 seconds of dissociation. The receptor on the surface was regenerated using a 30 second injection of 3 M MgCl_2_. Observed signal between 180 and 200 seconds was averaged for each sample. Average response observed for multiple 5 nM IL-17A samples without any peptide was used as 100% signal for calculating % inhibition for each concentration of compound. The data were fit with Microcal Origin software (OriginLab, MA) to calculate IC_50_ for peptides.

### FRET Assay

The FRET signal of a Eu^3+^-labeled IL-17A donor and an Alexa Fluor 647 labeled IL-17RA acceptor was measured to monitor the interaction of IL-17A and IL-17RA. Maximal FRET was observed when IL-17A was bound to IL-17RA and diminished FRET was observed when IL-17A was separated from IL-17RA. The excitation of the donor at a wavelength of 320 nm triggers fluorescence at 615 nM and this in turn serves to excite the acceptor, which then fluoresces at a wavelength of 665 nm. The fluorescence at both 615 nm and 665 nm were measured and the ratio of 665 nm/615 nm was used to monitor the IL-17A/IL-17RA binding. Final assay concentrations were 1 nM biotinylated IL-17A labeled with 0.67 nM Europium-Streptavidin (Invitrogen), 6 nM IL-17RA-Fc fusion protein (R&D Systems) labeled with 1 nM Alexa Fluor 647 antibody (BioLegand) in a buffer containing 10 mM HEPES pH 7.4, 150 mM NaCl, 0.02% BSA, and 0.01% Tween 20. Peptides were tested using a half log dilution series of 11 concentrations. The IL-17A was incubated with the Europium-Streptavidin to 1.5X final assay concentration for one hour at room temperature. Peptides were prepared at 50X final concentration in 100% DMSO and 300 nl were added to a 384-well white assay plate (Greiner). 10 μl of the Eu^3+^-labeled IL-17A was added to the peptides and incubated at room temperature for one hour. During this pre-incubation of peptide and IL-17A, IL-17RA was incubated with Alexa Fluor 647 antibody to 3X final assay concentration at room temperature for one hour and then 5 μl of the 3X Alexa Fluor 647 labeled IL-17RA was added to the assay for a total volume of 15.3 μl and a final DMSO concentration of 2%. The plates were covered and incubated at room temperature for 3 hours. The FRET signal of the IL-17A/IL-17RA interaction was measured using an EnVision Multilabel plate reader (PerkinElmer). The peptide data was converted into % inhibition, using 0% (no HAP) and 100% inhibition (100 nM HAP) as controls. A four parameter logistic nonlinear regression model using the percent inhibition at each concentration was used to calculate an IC_50_ for each peptide.

### Protein crystallization and data collection

To crystallize Fab/IL-17A/HAP complex, 30 mM HAP in DMSO stock was added to the Fab/IL-17A complex to a final concentration of 1 mM. The complex was then screened for crystals using commercial screen kits using a sitting drop vapor diffusion format. Crystals of Fab/truncated IL-17A/HAP complex were obtained under condition of 0.02 mM CdCl_2_, 0.02 M MgCl_2_, 0.02 M NiCl_2_, 0.1 M NaOAc pH = 4.2–4.9, and 24–28% PEG MME 2000. Crystals of Fab/full length IL-17A covalent dimer/HAP complex were obtained under conditions of 0.1 M NaOAc, pH = 4.5 and 30% PEG MME 5000. Crystals were soaked briefly in cryo solutions of the mother liquor supplemented with 25% glycerol before flash cooled in liquid nitrogen. Crystal data sets were collected at APS IMCA 17ID beamline (Chicago, IL), processed with autoPROC^42^. Data collection statistics are listed in Supplementary Table 2.

### Structure determination and refinement

Fab/IL-17A/HAP complex structures were solved with molecular replacement method using the published FAN/IL-17A crystal structure (pdb code 2VXS), using program Phaser^43^. Structure refinements was carried out using program Buster^44^ and manual model building using program COOT^45^. Final refinement statistics are listed in Supplementary Table 2.

## Additional Information

**How to cite this article**: Liu, S. *et al*. Inhibiting complex IL-17A and IL-17RA interactions with a linear peptide. *Sci. Rep*. **6**, 26071; doi: 10.1038/srep26071 (2016).

## Supplementary Material

Supplementary Information

## Figures and Tables

**Table 1:** ELISA competition activity of peptide analogues of 1.

Compound #	Competition IC_50_(nM)	1	2	3	4	5	6	7	8	9	10	11	12	13	14	15
**1**	80	I	V	V	T	M	P	A	D	L	W	D	W	I	K	A
**2**	80	I	V	V	T	M	P	A	D	L	W	D	W	I	R	A
**3**	66	I	V	V	T	M	P	A	D	L	W	D	W	I	R	**K**
**4**	172	I	V	V	T	M	P	A	D	L	W	D	W	I	**A**	A
**5**	402	I	V	V	T	M	P	A	D	L	W	D	W	**A**	R	A
**6**	7451	I	V	V	T	M	P	A	D	L	W	D	**A**	I	R	A
**7**	474	I	V	V	T	M	P	A	D	L	W	**A**	W	I	R	A
**8**	524	I	V	V	T	M	P	A	D	L	**A**	D	W	I	R	A
**9**	640	I	V	V	T	M	P	A	D	**A**	W	D	W	I	R	A
**10**	4016	I	V	V	T	M	P	A	**A**	L	W	D	W	I	R	A
**11**	84	I	V	V	T	M	P	**A**	D	L	W	D	W	I	R	A
**12**	2651	I	V	V	T	M	**A**	A	D	L	W	D	W	I	R	A
**13**	11	I	V	V	T	**A**	P	A	D	L	W	D	W	I	R	A
**14**	195	I	V	V	**A**	M	P	A	D	L	W	D	W	I	R	A
**15**	5717	I	V	**A**	T	M	P	A	D	L	W	D	W	I	R	A
**16**	149	I	**A**	V	T	M	P	A	D	L	W	D	W	I	R	A
**17**	167	**A**	V	V	T	M	P	A	D	L	W	D	W	I	R	A
**18**	45	I	**H**	V	T	M	P	A	D	L	W	D	W	I	R	A
**19**	71	I	**Q**	V	T	M	P	A	D	L	W	D	W	I	R	A
**20**	117	I	**R**	V	T	M	P	A	D	L	W	D	W	I	R	A
**21**	31	I	**T**	V	T	M	P	A	D	L	W	D	W	I	R	A
**22**	522	I	**W**	V	T	M	P	A	D	L	W	D	W	I	R	A
**23**	64	I	**Y**	V	T	M	P	A	D	L	W	D	W	I	R	A
**24**	3	I	V	V	T	**I**	P	A	D	L	W	D	W	I	R	A
**25**	33	I	V	V	T	**L**	P	A	D	L	W	D	W	I	R	A
**26**	6	I	V	V	T	**V**	P	A	D	L	W	D	W	I	R	A
**27**	31	I	V	V	T	M	P	A	D	L	W	D	W	I	**M**	A
**28**	26	I	V	V	T	M	P	A	D	L	W	D	W	I	**N**	A
**29**	33	I	V	V	T	M	P	A	D	L	W	D	W	I	**Q**	A

All peptides are acetylated at their N-termini.

**Table 2:** Cell-based activity of amino acid deletions of 30.

Compound #	Cell IC_50_(nM)	1	2	3	4	5	6	7	8	9	10	11	12	13	14	15
**1**	370	I	V	V	T	M	P	A	D	L	W	D	W	I	K	A
**24**	18	I	V	V	T	I	P	A	D	L	W	D	W	I	R	A
**26**	137	I	V	V	T	L	P	A	D	L	W	D	W	I	R	A
**30**	17	I	H	V	T	I	P	A	D	L	W	D	W	I	N	K
**32**	>1000		H	V	T	I	P	A	D	L	W	D	W	I	N	K
**33**	>1000			V	T	I	P	A	D	L	W	D	W	I	N	K
**34**	>1000				T	I	P	A	D	L	W	D	W	I	N	K
**35**	12	I	H	V	T	I	P	A	D	L	W	D	W	I	N	
**36**	183	I	H	V	T	I	P	A	D	L	W	D	W	I		
**37**	>1000	I	H	V	T	I	P	A	D	L	W	D	W			

**Table 3:** Binding affinity/IC50 of HAP in orthogonal *in vitro* assays/primary keratinocyte cell assay.

Assay	K_D_/IC_50_ (nM)
SPR binding (N = 3)*: hIL-17A, hIL-17A/F, mIL-17A	2.6(±0.5), 4.6(±0.7), 30.1(±0.1)
SPR competition	0.9(±0.3)
FRET IL-17RA competition (N = 598)	0.8 ± 0.2
Human primary keratinocytes IC-8 (N = 9), IC-6 (N = 4), CCL-20 (N = 4)	151(±49), 136(±37), 253(±64)

^*^K_D_ = k_d_/k_a_. k_a_ (M^−1^s^−1^)and k_d_ (s^−1^): 2(±1) × 10^5^, 6(±5) × 10^−4^ (hIL-17A); 4.6(±0.7) × 10^5^, 2.1(±0.2) × 10^−3^ (hIL-17A/F); 2.7(±0.2) × 10^5^, 8.2(±0.8) × 10^−3^ (mIL-17A).
