# Supplementary material for: Inhibiting complex IL-17A and IL-17RA interactions with a linear peptide
Source: Sci Rep. 2016 May 17;6:26071. doi: 10.1038/srep26071 (PMC4869123; doi:10.1038/srep26071)
Supplement: Supplementary Information [file srep26071-s1.pdf]

Supporting Information for Publication

Inhibiting complex IL-17A and IL-17RA interactions with a linear peptide

Shenping Liu, Joel Desharnais, Parag V. Sahasrabudhe, Ping Jin, Wei Li, Bryan D. Oates, Suman Shanker, Mary Ellen Banker, Boris B. Chrunyk, Xi Song, Xidong Feng, Matt Griffor, Judith Jimenez, Gang Chen, David Tumelty, Abhijit Bhat, Curt W. Bradshaw, Gary Woodnutt, Rodney W. Lappe, Atli Thorarensen, Xiayang Qiu, Jane M. Withka and Lauren D. Wood

Contents of supporting information:

Supplementary Methods

Scheme S1: Representative example of peptide dimer synthesis: synthesis of peptide **45**

Scheme S2: Synthesis of peptide **48**

Scheme S3: Sequences of non-commercial protein constructs used

Figure S1. Flow chart of identification of anti-IL-17A peptides

Figure S2. Inhibition of IL-17A induced IL-6 production by HAP in BJ fibroblasts

Figure S3. Characterizations for peptide **45**

Figure S4: Inhibition of IL-17A induced KC production by HAP and **45** in MLE-12 mouse epithelial cells

Figure S5. IL-17A and TNF-α synergistically stimulate production of pro-inflammatory IL-8 by human keratinocytes.

Figure S6. In SPR experiments, HAP and IL-17A can bind to anti-IL-17A antibody simultaneously

Figure S7. Evolving of the initial peptide **1** to HAP and **45**

Figure S8. Determination of IL-17A/HAP complex stoichiometry by Native Electrospray Ionization (ESI) Mass Spectrometry

Table S1. RP-HPLC-MS retention times and masses found

Table S2. In vitro cell activity of dimeric peptides and their analogues.

Table S3. X-ray data collection and structure refinement statistics

Methods

Protein production

Sequences of all non-commercial protein constructs used in this study are listed in Scheme 3S.

The N-terminal BAP and His_6 double tagged human IL-17A protein used in SPR and FRET assay was expressed in HEK293 cells as a secreted protein, using the FreeStyleTM MAX transfection kit (Invitrogen). The culture media was collected by centrifugation and filtered through a 0.22-μm filter. The protein was purified on a TALON metal affinity column using an imidazole gradient in 50 mM Hepes pH 7.5, 500 mM NaCl, followed by a size exclusion chromatography on a Superdex75 column (GE Healthcare) in the final size exclusion buffer of phosphate saline buffer supplemented with 150 mM NaCl. *In vitro* biotinylation of the protein was done at protein concentration of 1.3 mg/ml using the BirA Biotin Ligase kit (Avidity, Colorado, US) following the suggested protocol. The reaction mixture was incubated at 30°C for 6 hours. The biotinylated protein was further cleaned with size exclusion using Superdex 75 column. Main protein fractions were collected and biotinylation was confirmed with Mass Spectrometry.

The C-terminal-Protein C-His6-BAP tagged IL-17F used in SPR binding assay was expressed in HEK293F cells as a secreted protein and purified using protein C affinity chromatography followed by size exclusion, and was *in vitro* biotinylated as described above.

The IL-17A/F heterodimer used in SPR consists of the C-terminal-Protein C-His6-Bap tagged IL-17F and the C-terminal FLAG tagged IL-17A. The heterodimer was co-expressed using both constructs at equal amount in HEK293 cells. The protein was purified using anti-FLAG affinity chromatography, followed by protein C affinity chromatography and size exclusion. The heterodimer was biotinylated as described above.

The truncated IL-17A used in co-crystallization was produced based on published structure[1], except the protein was expressed in baculovirus-insects system. The protein was purified on a His Trap Excel metal affinity column and eluted with an imidazole gradient. The protein was further purified on a Superdex75 column in the size exclusion buffer.

The full length protein with the mature IL-17A sequence use in crystallization was expressed in HEK293 cells. The protein was purified on a His Trap Excel metal affinity column followed by size exclusion chromatography. The fractions from size exclusion were pooled and de-glycosylated by treating with PNGase-F over night at 4°C. The full length protein was further purified to separate the disulfide bonds cross linked IL-17A dimer from the non-covalent dimer by Reversed Phase HPLC on a Xbridge C8, 19x100 mm, 5 micron column (Waters). The fractions from size exclusion were pooled and de-glycosylated by treating with PNGase-F over night at 4°C. The Reversed Phase solvents used were A: 0.1% TFA, 2% acetonitrile in water and B, 100% acetonitrile. A very shallow gradient of 0.5% B/min was applied. The fractions were analyzed by LCMS for purity and the covalent dimer fractions were pooled and dried down by rotary evaporation to remove solvents. The dried protein was re-dissolved in the size exclusion buffer.

Alternatively, the full length mature IL-17A used in co-crystallization was expressed in E. coli and refolded from inclusion bodies[2]. The refolded protein was purified by size exclusion chromatography and subjected to RP-HPLC for separation of IL-17A covalent dimer and the dissociable dimer as described above.

The anti-IL-17A antibody FAB fragment used for co-crystallization was designed based on the published crystal structure of an IL-17A/FAB complex[3]. A His_6 tag was placed at the N-terminus of the heavy chain of the FAB to facilitate affinity purification. The FAB heavy and light chains were co-expressed in HEK293 cells (AkesoBio, China), and purified on a TALON metal affinity column, followed by size exclusion on a Superdex200 column.

The FAB complex with the truncated IL-17A or the full length covalent linked IL-17A dimer was generated by mixing IL-17A dimer with slightly over 2 folds of molar ratio amount of FAB. The IL-17A/FAB complex was separated from the free FAB by size exclusion on a Superdex 200 column. The fractions containing the IL-17A/FAB complex were concentrated to 8-9mg/ml for crystallization.

Native electrospray ionization mass spectrometry

Purified full length disulfide bond linked IL-17A dimer expressed in mammalian cells (1.2 mg/ml) was buffer exchanged into 200 mM ammonium acetate buffer (pH 7.5) using Amicon Ultra 0.5 ml centrifugal filters (Millipore) and then incubated with HAP at different molar ratios. A few µl aliquots were electrosprayed via the NanoLockSpray ion source (Waters, Milford, MA) using Pico Tip Glass Tips (New Objective, Woburn, MA). Mass spectra were recorded on a hybrid Synapt G2 Q-ToF instrument (Waters) with instrument conditions optimized to preserve non-covalent interactions[4]. The following were a few typical instrumental parameters particularly important for preserving noncovalent interaction: backing pressure ~6 millibars, capillary voltage ~1.2 kV, sampling cone voltage ~90 eV, and trap collision energy 5 eV. Mass spectra were acquired and analyzed using MassLynx 4.1.1 software (Waters).

References

1 Liu, S. et al. Crystal structures of interleukin 17A and its complex with IL-17 receptor A. Nat Commun 4, 1888, doi:10.1038/ncomms2880 (2013).

2 Wu, B. et al. The role of interchain disulfide bond in a recombinant human interleukin-17A variant. Cytokine 65, 167-174, doi:10.1016/j.cyto.2013.11.007 (2014).

3 Gerhardt, S. et al. Structure of IL-17A in Complex with a Potent, Fully Human Neutralizing Antibody. Journal of Molecular Biology 394, 905-921, doi:http://dx.doi.org/10.1016/j.jmb.2009.10.008 (2009).

4. Hernández, H., and Robinson, C. V. (2007) Determining the stoichiometry and interactions of macromolecular assemblies from mass spectrometry. Nat. Protoc. 2, 715–726

Representative example of peptide dimer synthesis: synthesis of peptide 45

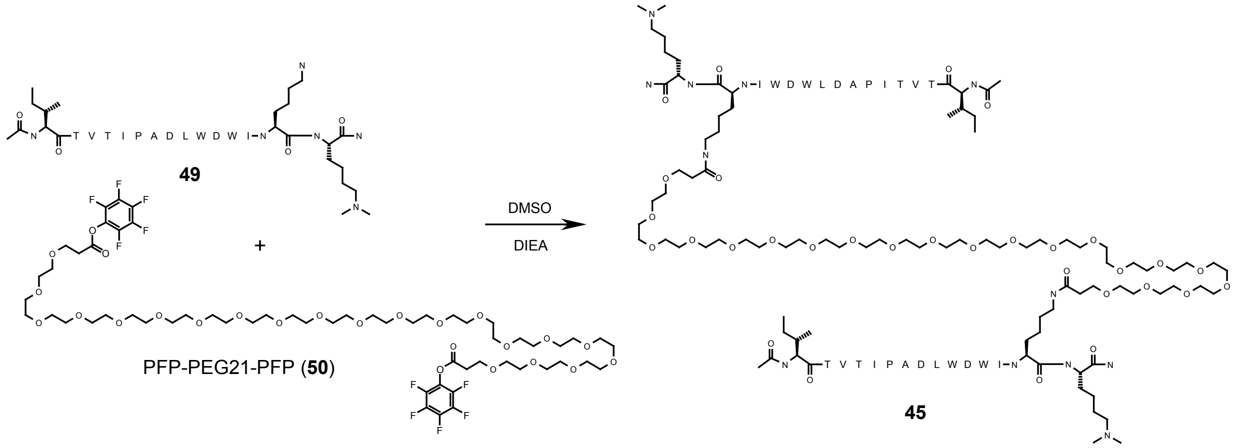

Scheme S1.

Peptide having the sequence Ac-ITVTIPADLWDWIKK(Me)$_2$-NH$_2$ (**49**, 273 mg, 146 mmmol) was dissolved in DMSO (6 mL). Diisopropylethylamine (DIEA, 0.15 mL, 438 mmol) was added and the mixture was stirred at r.t. for 5 min. The solution was transferred to a vial containing the PEG21 bispentafluoroester (**50**, 80 mg, 58 mmol) and the solution was stirred at r.t. for 1 hr. The mixture was directly injected into a Waters preparative HPLC for purification and the desired fractions were pooled and lyophilized resulting in 206 mg of white powder (43 mmol, 74%).

Synthesis of peptide 48

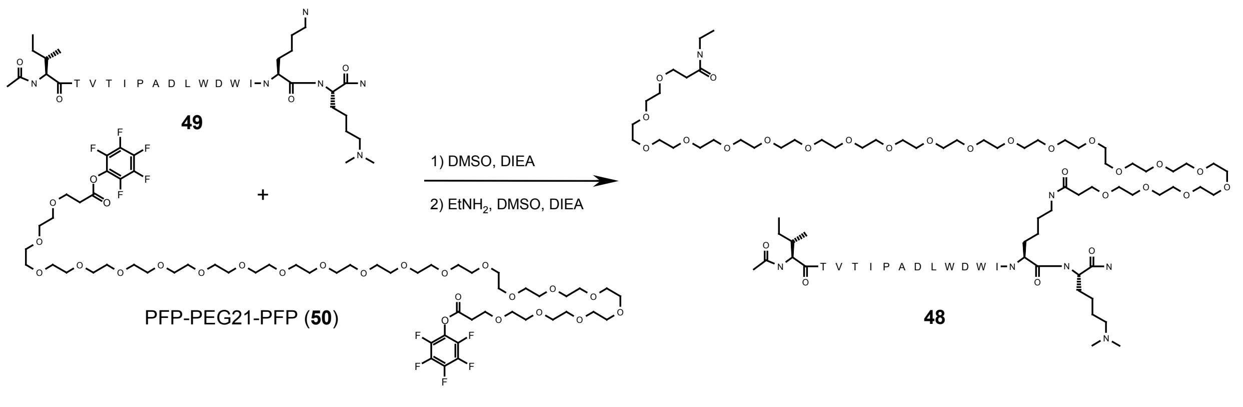

Scheme S2.

Peptide having the sequence Ac-ITVTIPADLWDWIKK(Me)$_2$-NH$_2$ (**49**, 50 mg, 27 mmmol) was dissolved in DMSO (5 mL). DIEA (23 μL, 134 mmol) was added and the mixture was stirred at r.t. for 5 min. The solution was transferred to a vial containing the PEG21 bispentafluoroester (**50**, 82 mg, 59 mmol) and the solution was stirred at r.t. for 1 hr. The mixture was directly injected into a Waters preparative HPLC for purification and the desired fractions were pooled and lyophilized. The resulting solid was dissolved in DMSO (5 mL). Ethylamine (18 μL, 270 mmol) and DIEA (47 μL, 270 mmol) were added and the mixture was stirred at r.t. for 1 hr. The mixture was directly injected into a Waters preparative HPLC for purification and the desired fractions were pooled and lyophilized resulting in 21.9 mg of white powder (7.5 mmol, 28%).

1) GSHHHHHHGSENLYFQGEVQLLESGGGLVQPGGSLRLSCAASGFTFSSYAMS
WVRQAPGKGLEWVSAISGSGGSTYYADSVKGRFTISRDNSKNTLYLQMNSLRAEDTAVYYCARDLIH
GVTRNWGQGTLVTVSSASTKGPSVFPLAPSSKSTSGGTAALGCLVKDYFPQPVTVSWNSGALTSGVH
TFPAVLQSSGLYSLSSVVTVPSSSLGTQTYICNVNHKPSNTKVDKKVEPKS

2) NFMLTQPHSVSESPGKTVTISCTRSSGSLANYYVQWYQQRPGSSPTIVIFANN
QRPSGVPDRFSGSIDSSSNSASLTISGLKTEDEADYYCQTYDPYSVVFGGGTKLTVLGQPKAAPSVTL
FPPSSEELQANKATLVCLISDFYPGAVTVAWKADSSPVKAGVETTTPSKQSNNKYAASSYLSLTPEQW
KSHRSYSCQVTHEGSTVEKTVAPTE

3) DPNSEDKNFPRTVMVNLNIHNRNTNTNPKRSSDYYDRSTSPWNLHRNEDPE
RYPSVIWEAKCRHLGCINADGNVDYHMNSVPIQQEILVLRREPPHSPNSFRLEKILVSVGCTCVTPIVH
HVA

4) DPGLNDIFE AQKIEWHEGS GHHHHHHGSG IVKAGITIPR NPGCPNS EDK NFPRTVMVNL
NIHNRNTNTN PKRSSDYYNR STSPWNLHRN EDPERYPSVI WEAKCRHLGC INADGNVDYH
MNSVPIQQE LVLRREPPHC PNSFRLEKIL VSVGCTCVTP IVHHVA

5) DPIVKAGITIPRNPGCPNSEDKNFPRTVMVNLNIHNRNTNTNPKRSSDYYNR
STSPWNLHRNEDPERYPSVIWEAKCRHLGCINADGNVDYHMNSVPIQQEILVLRREPPHCPNSFRLE
KILVSVGCTCVTPIVHHVA

6) DPRKIPKVGHTFFQKPESSPPVPGGSMKLDIGIINENQRVSMSRNIESRSTS
PWNYTVTWDPNRYPSEVVQAQCRNLGCINAQGKEDISMNSVPIQQETLVVRRKHQGSSVSFQLEKV
LVTVGCTCVTPVIHHVQENLYFQGEDQVDPRLIDGKHHHHHHGAGLNDIFEAQKIEWHE

7) DPGIVKAGITIPRNPGSPNSEDKNFPRTVMVNLNIHNRNTNTNPKRSSDYY
NRSTSPWNLHRNEDPERYPSVIWEAKCRHLGCINADGNVDYHMNSVPIQQEILVLRREPPHSPNSFR
LEKILVSVGCTCVTPIVHHVAENLYFQGDYKDDDDK

8)GITIPRNPGCPNSEDKNFPRTVMVNLNIHNRNTNTNPKRSSDYYNRSTSPWNLHRNEDPERYPSVI
WEAKCRHLGCINADGNVDYHMNSVPIQQEILVLRREPPHCPNSFRLEKILVSVGCTCVTPIVHHVA

Scheme S3. Sequences of non-commercial proteins used in this study

1) N-His tagged FAB heavy chain used for co-crystallization 2) FAB light chain used in co-crystallization 3) N-terminus truncated IL-17A used for co-crystallization. 4) BAP-His tagged full length IL-17A used in SPR and FRET 5) Full length IL-17A used in crystallization and native mass spectrum analysis 6) C-terminal His-BAP tagged IL-17F in IL-17FF or IL-17AF heterodimer used in SPR 7)C-terminal FLAG tagged IL-17A in IL-17AF heterodimer used in SPR 8) Full length IL-17A expressed in E.coli for refolding work.

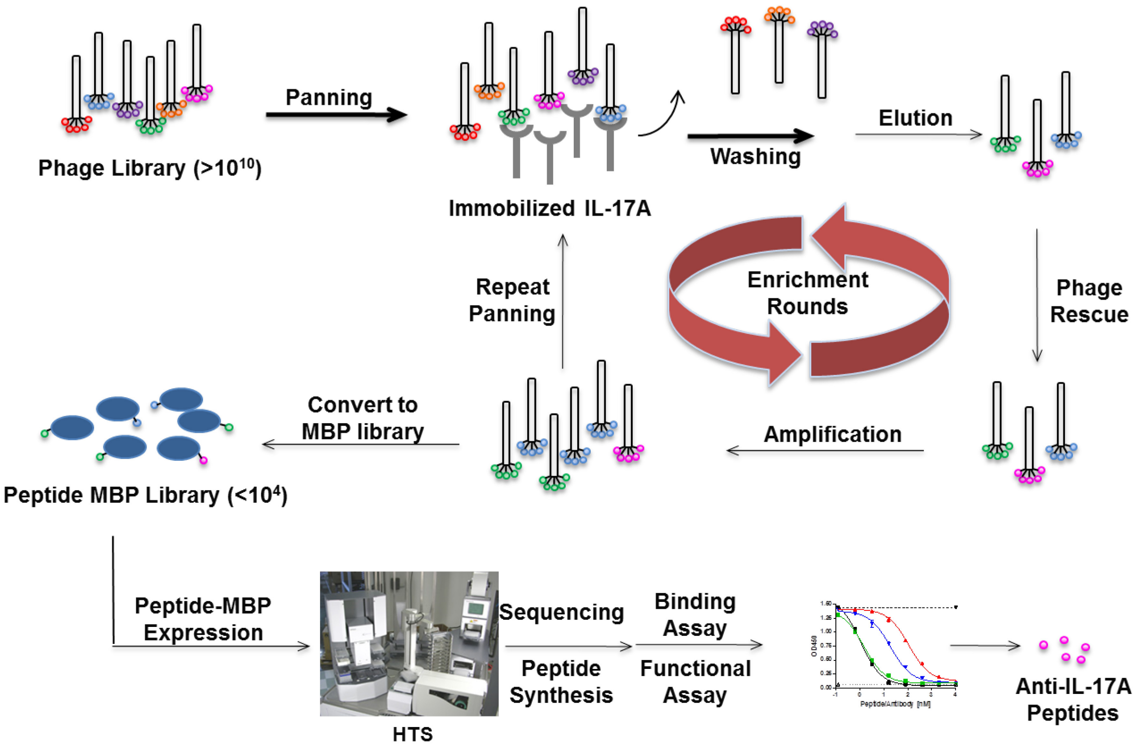

Figure S1. Flow chart of identification of anti-IL-17A peptides.

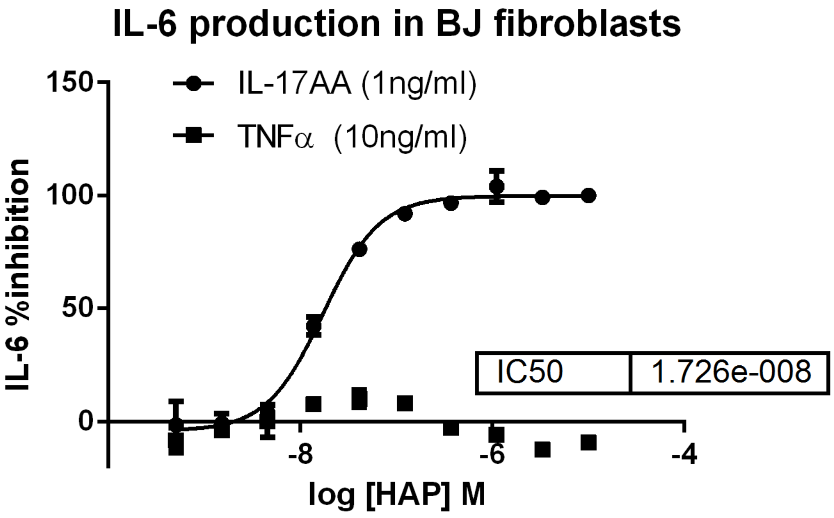

Figure S2. Inhibition of IL-17A induced IL-6 production by HAP in BJ fibroblasts. BJ cells were cultured in the presence of TNF-α (10 ng/ml) or IL-17A (1 ng/ml) with a series of concentration of HAP for 24 hours after which time cell culture supernatants were removed for analysis of IL-6 production. It is clear that HAP does not inhibit TNF-α induced IL-6 production.

Sample Report:

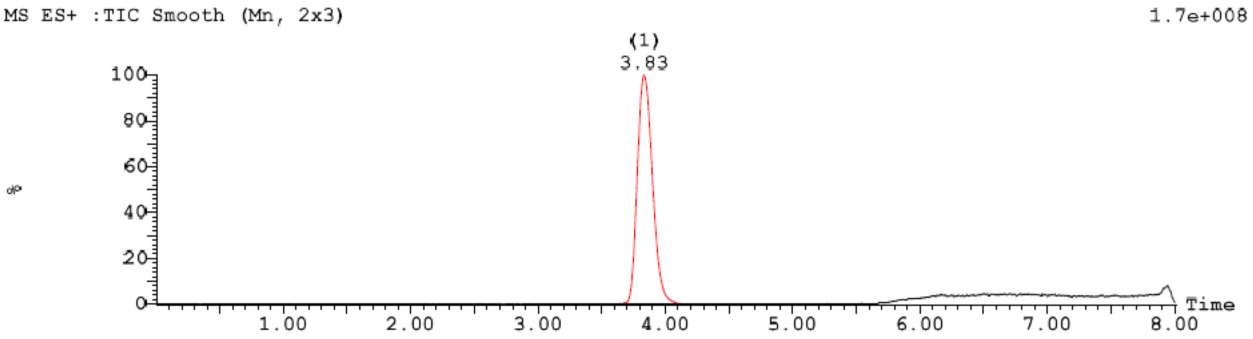

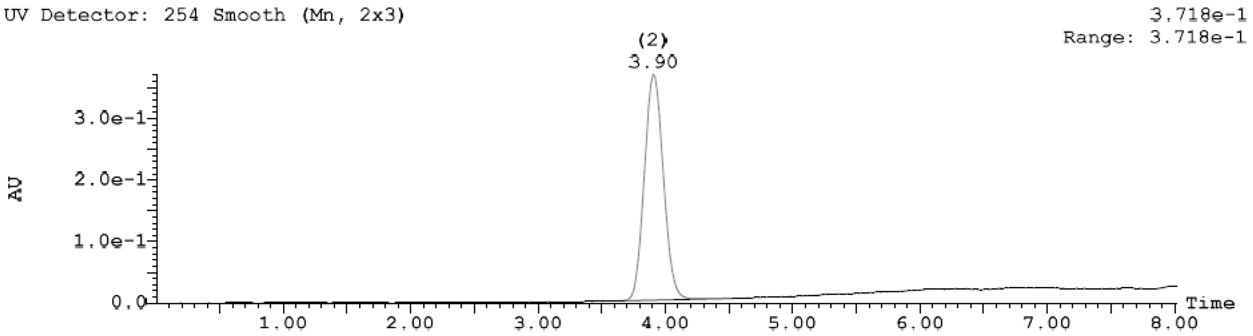

Peak Number	Compound	Time	AreaAbs	Area %Total	Width	Mass Found
2		3.90	63586	100.00	1	

Peak ID	Compound	Time	Mass Found	Area %BP
1		3.83		100

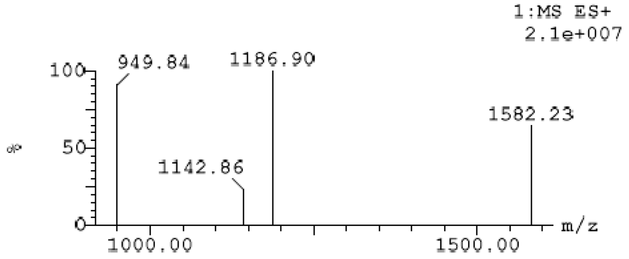

Figure S3. Characterizations of peptide **45**.

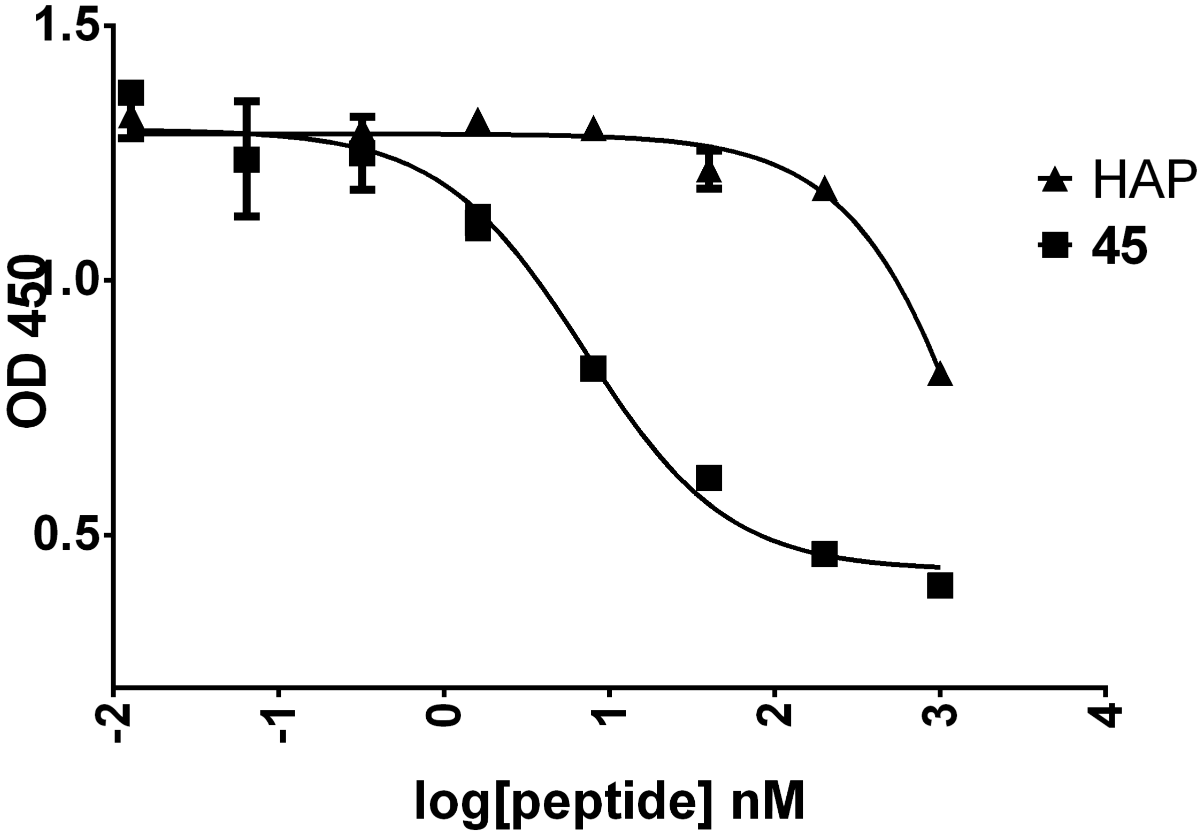

Figure S4. Inhibition of IL-17A induced murine keratinocyte-derived cytokine (KC). MLE-12 mouse epithelial cells were cultured in the presence of IL-17A (15 ng/ml) with a series of concentration of **45** and HAP for 24 hours after which time cell culture were removed for analysis of KC production**.** Data are mean and error bars of +/- standard deviation of duplicated measurements

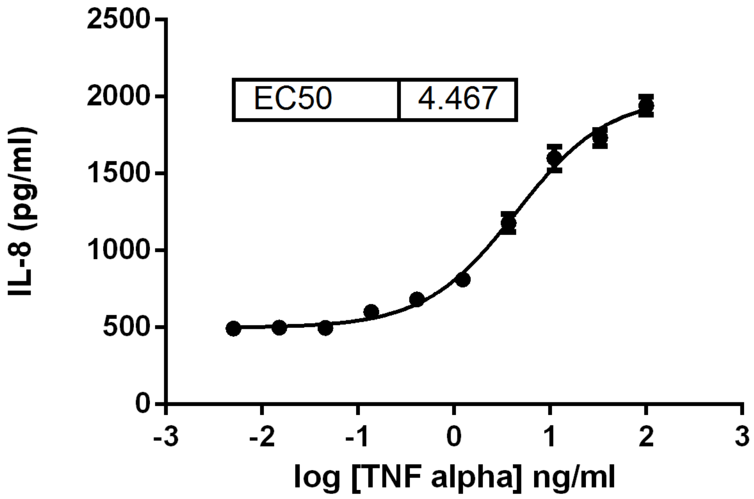

A

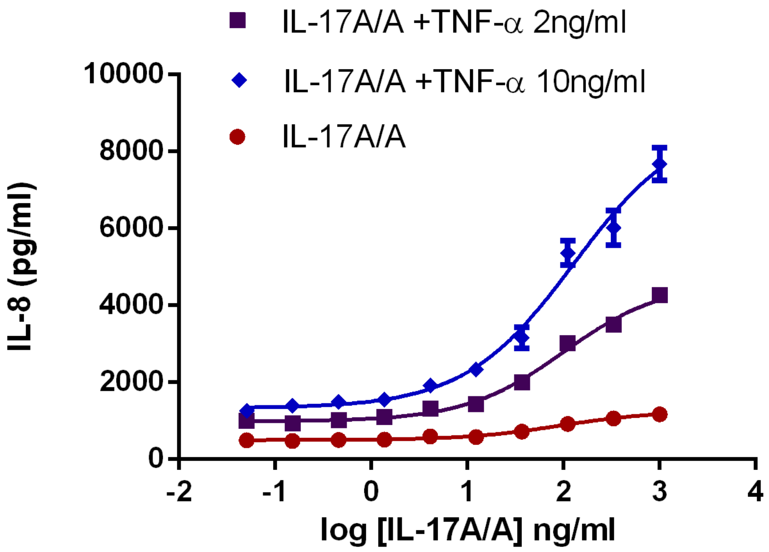

B

Figure S5. IL-17A and TNF-α synergistically stimulate production of pro-inflammatory IL-8 by human keratinocytes. Data are mean and error bars of +/- standard deviation of three measurements. A. Dose responsive curve of IL-8 production stimulated by TNF-α alone. Dose responsive curves of IL-8 production stimulated by IL-17A in the presence of TNF-α at indicated concentrations.

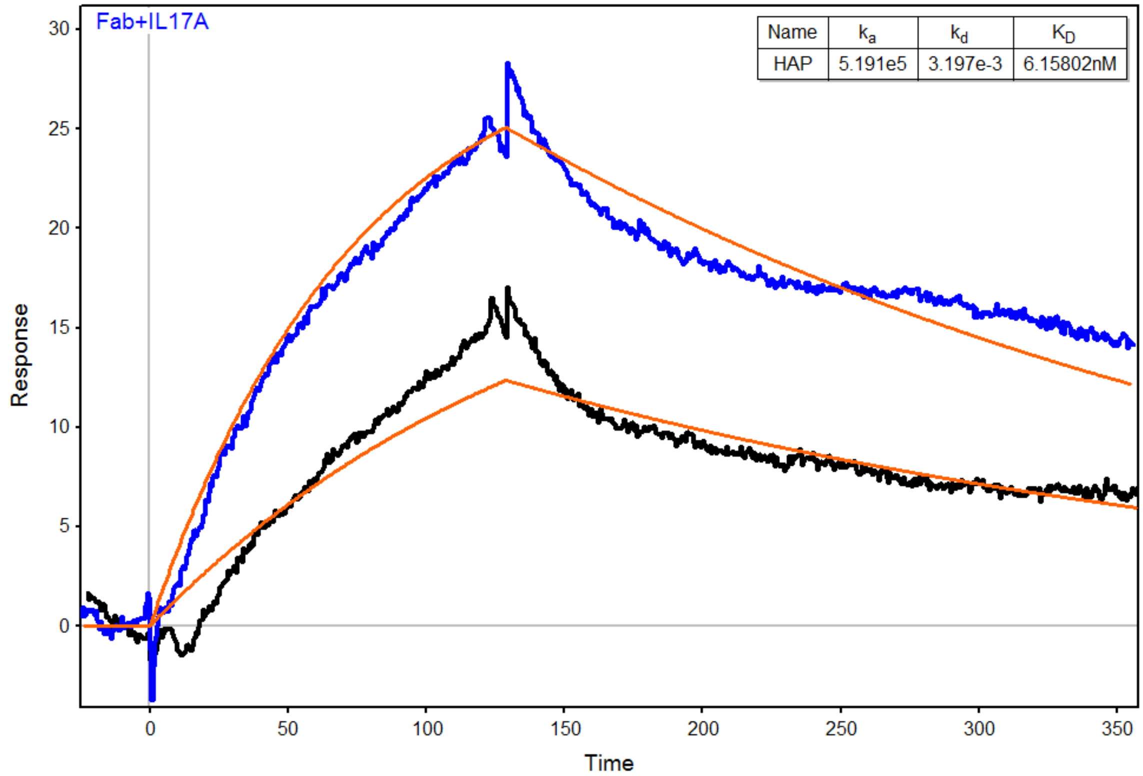

Figure S6. HAP binding to IL-17A:Fab complex. Chemically biotinylated Fab was captured on SA chip to about 3000 RU. IL-17A (Cell Signaling) at 200 nM was injected to bind about 600 RU of IL-17A. 7 nM and 20 nM HAP were injected in separate cycles after regenerating the Fab surface with 3 M $MgCl_2$ and reloading with an injection of 200 nM IL-17A. HAP binds to IL-17A complexed with Fab with a K_D of about 6 nM which is comparable to HAP binding to IL-17A alone (K_D = 2 nM).

HAP Ac-I**H**VTIPADLWDWINK
45 Ac-I**T**VTIPADLWDWIKK(Me2)-PEG21-repeat
1 Ac-I**V**VT**M**PADLWDWIKA

Figure S7. Evolving of the initial peptide **1** to HAP and **45**. Cellular activies (BJ human fibroblast) of these peptides: 370, 31 and 0.1 nM, respectively. The amphipathic nature of the HAP β-strand bound to IL-17A explains the preference of the hydrophilic residues at the 2 and 4 positions of peptides.

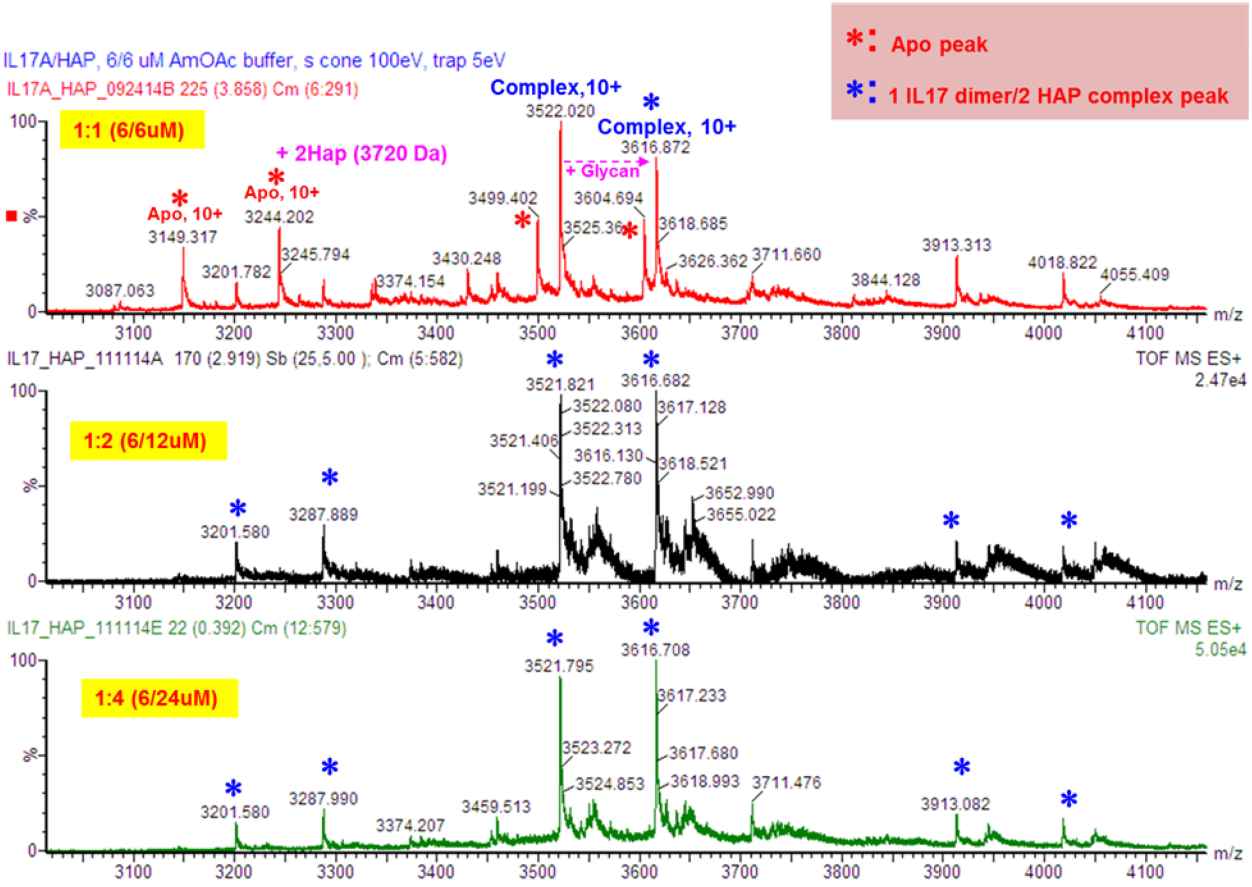

Figure S8. Determination of HAP/IL-17A complex stoichiometry using native Electrospray Ionization (ESI) Mass Spectrometry (MS). With increasing amount HAP, either free IL-17A dimer and IL-17A dimer/2 HAP complex were detected (Top), or only IL-17A dimer/2 HAP complex was detected (middle and bottom).

Compound #	Formula	Peptide MW (calculated)	Peptide MW (measured)	Mass Accuracy (ppm)	Retention Time (min)	HPLC Method	Purity (UV254) %
**1**	C86H131N19O21S	1797.9488	1797.9322	9.23	8.696	1	95
**2**	C86H131N21O21S	1825.9549	1825.946	4.87	8.999	1	95
**3**	C89H138N22O21S	1883.0128	1882.9998	6.90	9.318	1	95
**4**	C83H124N18O21S	1740.8909	1740.8986	4.42	9.929	1	95
**5**	C83H125N21O21S	1783.908	1783.9018	3.48	9.049	1	95
**6**	C78H126N20O21S	1710.9127	1710.9056	4.15	8.797	1	95
**7**	C85H131N21O19S	1781.9651	1781.9654	0.17	9.254	1	95
**8**	C78H126N20O21S	1710.9127	1710.9056	4.15	8.901	1	95
**9**	C83H125N21O21S	1783.908	1783.9018	3.48	8.866	1	95
**10**	C85H131N21O19S	1781.9651	1781.9654	0.17	8.833	1	95
**11**	C89H138N22O21S	1883.0128	1882.9998	6.90	9.26	1	95
**12**	C84H129N21O21S	1799.9393	1799.9298	5.28	10.215	1	93
**13**	C84H127N21O21	1765.9515	1765.9376	7.87	9.15	1	95
**14**	C85H129N21O20S	1795.9443	1795.9358	4.73	9.315	1	95
**15**	C84H127N21O21S	1797.9236	1797.906	9.79	9.286	1	93
**16**	C84H127N21O21S	1797.9236	1797.906	9.79	9.297	1	93
**17**	C83H125N21O21S	1783.908	1783.9018	3.48	9.511	1	95
**18**	C87H129N23O21S	1863.9454	1863.9302	8.15	9.025	1	95
**19**	C86H130N22O22S	1854.9451	1854.9522	3.83	9.268	1	95
**20**	C87H134N24O21S	1882.9876	1882.9728	7.86	8.791	1	95
**21**	C85H129N21O22S	1827.9342	1827.9362	1.09	9.248	1	95
**22**	C92H132N22O21S	1912.9658	1912.9646	0.63	9.801	1	95
**23**	C90H131N21O22S	1889.9498	1889.9436	3.28	9.378	1	95
**24**	C87H133N21O21	1807.9985	1807.9884	5.59	8.041	1	95
**25**	C87H133N21O21	1807.9985	1807.9884	5.59	8.134	1	95
**26**	C86H131N21O21	1793.9828	1793.972	6.02	7.711	1	95
**27**	C85H128N18O21S2	1800.8943	1800.8814	7.16	8.799	1	95
**28**	C84H125N19O22S	1783.8967	1783.8814	8.58	8.011	1	95
**29**	C84H125N19O22S	1783.8967	1783.8814	8.58	8.054	1	95
**30**	C89H132N22O22	1860.9887	1860.9866	1.13	3.52	2	95
**31**	C87H130N22O21	1818.9781	1818.966	6.65	3.35	2	95
**32**	C83H121N21O21	1747.9046	1747.8868	10.18	8.117	1	95
**33**	C77H114N18O20	1610.8457	1610.8518	3.79	8.734	1	95
**34**	C72H105N17O19	1511.7773	1511.7596	11.71	8.217	1	95
**35**	C83H120N20O21	1732.8937	1732.8922	0.87	8.389	1	95
**36**	C79H114N18O19	1618.8508	1618.846	2.97	8.358	1	95
**37**	C73H103N17O18	1505.7667	1505.7652	1.00	8.27	1	95
**38**	C89H136N20O23	1853.0087	1853.0022	3.51	3.7	2	95
**39**	C220H354N40O67	4628.5523	4628.5464	1.27	3.85	2	95
**40**	C196H304N42O51	4062.2486	4062.2598	2.76	3.84	2	95
**41**	C228H368N42O67	4766.668	4766.6172	10.66	3.79	2	95
**42**	C198H308N42O53	4122.2697	4122.2583	2.77	3.8	2	95
**43**	C231H376N42O69	4826.6891	4826.6984	1.93	3.84	2	95
**44**	C196H306N40O51	4036.2581	4036.233	6.22	3.95	2	95
**45**	C228H370N40O67	4740.6775	4740.6388	8.16	3.9	2	95
**46**	C204H318N44O55	4264.3439	4264.3104	7.86	3.75	2	95
**47**	C236H382N44O71	4968.7634	4968.7635	0.02	3.75	2	95
**48**	C139H235N21O45	2918.6746	2918.6451	10.11	3.79	2	95
**49**	C91H142N20O22	1867.0608	1867.0616	0.43	3.67	2	95

Table S1. RP-HPLC-MS retention times and masses found of synthesized peptides.

Compound #	Cell IC_{50} (nM)		1	2	3	4	5	6	7	8	9	10	11	12	13	14	15	
**38**	12		I	T	V	T	I	P	A	D	L	W	D	W	I	N	$K(Me)_2$	
**39**	0.74	(PEG21)	I	T	V	T	I	P	A	D	L	W	D	W	I	N	$K(Me)_2$	
**40**	3370		I	T	V	K(PEG5)	I	P	A	D	L	W	D	W	I	N	$K(Me)_2$	
**41**	15		I	T	V	K(PEG21)	I	P	A	D	L	W	D	W	I	N	$K(Me)_2$	
**42**	24		I	T	V	T	I	P	K(PEG5)	D	L	W	D	W	I	N	$K(Me)_2$	
**43**	1.4		I	T	V	T	I	P	K(PEG21)	D	L	W	D	W	I	N	$K(Me)_2$	
**44**	4.3		I	T	V	T	I	P	A	D	L	W	D	W	I	K(PEG5)	$K(Me)_2$	
**45**	0.1		I	T	V	T	I	P	A	D	L	W	D	W	I	K(PEG21)	$K(Me)_2$	
**46**	582		I	T	V	T	I	P	A	D	L	W	D	W	I	N	$K(Me)_2$	K(PEG5)
**47**	16		I	T	V	T	I	P	A	D	L	W	D	W	I	N	$K(Me)_2$	K(PEG21)
**48**	21		I	T	V	T	I	P	A	D	L	W	D	W	I	K(PEG21)*	$K(Me)_2$	

Table S2. In vitro cell activity of dimeric peptides and their analogues.

Crystal	FAB/truncated IL-17A/HAP	FAB/full length IL-17A/HAP
PDB code	5HHV	5HHX
Resol (Å).	95.-2.2 (2.46-2.2)	98-3.0 (3.35-3.0)
Space group	P321	P321
Unit cell	110.20 110.20 90.3	113.40 113.40 86.79
	90 90 120	90 90 120
No. Obs	265,655 (72,635)	125,304 (3,116)
No. Ref.	32,509 (9,127)	13,244 (3,698)
Rsym(%)	9.5 (149)	13.4 (115)
I/σ	14.7 (1.5)	13.4 (2.1)
Compl. (%)	100 (99.9)	99.9 (99.9)
Redundancy	8.2 (8.0)	9.5 (9.8)
Rfactor(%)	19.8 (24.0)	17.6 (22.9)
Rfree(%)	23.5 (27.9)	26.3 (30.2)

Table S3. X-ray crystallographic data collection and refinement statistics. Numbers in the parenthesis are in the highest resolution shells.
